# Mean dose rate in ultra-high dose rate electron irradiation is a significant predictor for O_2_ consumption and H_2_O_2_ yield

**DOI:** 10.1088/1361-6560/ace877

**Published:** 2023-08-07

**Authors:** Jacob P Sunnerberg, Rongxiao Zhang, David J Gladstone, Harold M Swartz, Jiang Gui, Brian W Pogue

**Affiliations:** 1 Thayer School of Engineering at Dartmouth College, Hanover, NH, United States of America; 2 Dartmouth-Hitchcock Medical Center, Lebanon, NH, United States of America; 3 Geisel School of Medicine at Dartmouth College, Hanover, NH, United States of America; 4 University of Wisconsin—Madison, Madison, WI, United States of America

**Keywords:** FLASH, ultra-high dose rate, oxygen, reactive oxygen species, dose rate, radiotherapy

## Abstract

*Objective*. The objective of this study was to investigate the impact of mean and instantaneous dose rates on the production of reactive oxygen species (ROS) during ultra-high dose rate (UHDR) radiotherapy. The study aimed to determine whether either dose rate type plays a role in driving the FLASH effect, a phenomenon where UHDR radiotherapy reduces damage to normal tissues while maintaining tumor control. *Approach*. Assays of hydrogen peroxide (H_2_O_2_) production and oxygen consumption (ΔpO_2_) were conducted using UHDR electron irradiation. Aqueous solutions of 4% albumin were utilized as the experimental medium. The study compared the effects of varying mean dose rates and instantaneous dose rates on ROS yields. Instantaneous dose rate was varied by changing the source-to-surface distance (SSD), resulting in instantaneous dose rates ranging from 10^2^ to 10^6^ Gy s^−1^. Mean dose rate was manipulated by altering the pulse frequency of the linear accelerator (linac) and by changing the SSD, ranging from 0.14 to 1500 Gy s^−1^. *Main results*. The study found that both ΔH_2_O_2_ and ΔpO_2_ decreased as the mean dose rate increased. Multivariate analysis indicated that instantaneous dose rates also contributed to this effect. The variation in ΔpO_2_ was dependent on the initial oxygen concentration in the solution. Based on the analysis of dose rate variation, the study estimated that 7.51 moles of H_2_O_2_ were produced for every mole of O_2_ consumed. *Significance*. The results highlight the significance of mean dose rate as a predictor of ROS production during UHDR radiotherapy. As the mean dose rate increased, there was a decrease in oxygen consumption and in H_2_O_2_ production. These findings have implications for understanding the FLASH effect and its potential optimization. The study sheds light on the role of dose rate parameters and their impact on radiochemical outcomes, contributing to the advancement of UHDR radiotherapy techniques.

## 1. Introduction

One of the most important questions in the emerging field of ultra-high dose rate (UHDR) radiotherapy is the underlying cause of the observed normal tissue sparing, or FLASH effect, seen in animal models (Favaudon *et al* 2014). It seems plausible that the production of reactive oxygen species (ROS) (Montay-Gruel *et al* 2019) and/or the change in local oxygen partial pressure (ΔpO_2_) presence (Cao *et al* 2021, Jansen *et al* 2021, El Khatib *et al* 2022) have significant roles given the well-known dominance of oxygen effect in radiobiological damage. To gain fuller understanding, it is important to consider crucial physical parameters of the delivery of radiation including mean dose rate, instantaneous dose rate, pulse structure and time of irradiation (Ashraf *et al* 2020, Folkerts *et al* 2020, Schuler *et al* 2022). In this study, the two dose rate parameters, mean and per-pulse, were specifically examined for their effect upon ROS, to compare their relative contributions to the underlying radiochemistry. It is possible to independently vary mean dose rate by pulse frequency, and then also vary mean and per-pulse dose rate by changing the distance between source to sample, thereby being able to determine if they were independent or linked drivers of ROS production.

The interaction of primary radicals from water radiolysis with DNA is thought to be responsible for the dominant fraction of strand break damage (Hirayama *et al* 2013, Favaudon *et al* 2022) and the subsequent ROS also contribute to damage. However, the relative contributions of these various species are not well-characterized and likely vary with the biological system as well as the radiation modality. Still, a dominant theory of the mechanism for the FLASH effect from UHDR is related to reduced yields of these damage-causing species in normal tissues (Favaudon *et al* 2022). Due to the short-lived and reactive nature of many of the potential oxidative species, they are difficult to measure *in vivo*. It is therefore relevant to quantify the yields in various *in vitro* protein environments. A production of one potentially important ROS, which has a relatively long life-time and therefore can be conveniently measured, hydrogen peroxide, has been shown to be reduced by increased dose rates (Montay-Gruel *et al* 2019, Blain *et al* 2022). This has been used as a surrogate for ROS measurements because it is a long-lived product of radiolysis and can be assayed without the need for fast transient spectroscopy methods.

Previous *in vitro* and *in vivo* studies using electron (Cao *et al* 2021) and proton (El Khatib *et al* 2022, Van Slyke *et al* 2022) beamlines have shown a reduction in the oxygen partial pressure consumed (ΔpO_2_) per-Gy of dose, for UHDR compared to conventional irradiations. In both studies, *in vitro* experiments resulted in lower consumption rates (O_2_ consumed per unit dose) with UHDR compared to conventional. Cao *et al* used Oxyphor 2P and Van Slyke *et al* both used Oxyphor PtG4 to measure changes in pO_2_ by phosphorescence quenching. The decrease in consumption, or ΔpO_2_, is an indicator of a reduction in oxygen lost due to its combination with other radical species to form peroxyl radicals. So, decreases in ΔpO_2_ may be interpreted as indicating a decrease in radiochemical damaging species.

These assays provide a sensitive and accurate way to measure the relative importance of dosimetry factors, such as instantaneous or mean dose rate used in UHDR delivery, to potential mechanisms that may underly the FLASH effect. One dominant theory has been that the reactive species effects during UHDR pulses could have a temporal density that affects their re-combination or alters the frequency of reaction with certain biological molecules (Favaudon 2022). In this study, protein solutions with oxygen-sensitive or hydrogen peroxide-sensitive probes were irradiated under various instantaneous and mean dose rates to investigate the impact on radiochemical mechanisms *in vitro*. The diffusion coefficient of oxygen in pure water is approximately 2 × 10^−5^ cm^2^ s^−1^, and a slightly lower coefficient of the same order of magnitude has been reported in tissue (Thews 1960, Ganfield *et al* 1970, Clark *et al* 1977, Homer *et al* 1983). We anticipate the diffusion coefficient in our assay to be of the same order of magnitude as the coefficients in tissue and pure water, and not being a barrier to quantifying ROS on these longer timescales. The heterogeneity of oxygen diffusion has been a subject of substantial study over the years, and results indicate that significant gradients can exist across extracellular and intracellular environments (Jones 1986). This effect complicates any measurement and interpretation of oxygen and slower transients *in vivo*.

There are data suggesting that the instantaneous per-pulse dose rate is a key factor in achieving the FLASH effect for electron deliveries (Montay-Gruel *et al* 2021). Findings to date suggest that, for electron delivery, that requirements are of total irradiation time less than 1 s for 10 Gy and instantaneous dose rates exceeding 10^5^ Gy s^−1^ are required (figure 1 in Montay-Gruel *et al* (2021)). However, pencil-beam scanning proton radiotherapy studies have demonstrated FLASH effects when their maximum dose rates are well below 10^5^ Gy/s. This suggests that overall, it may be the mean dose rate and/or irradiation time that dictates the FLASH effect as both mean dose rate and delivery times are comparable for electron and proton UHDR. Thus, further studies are needed to determine the relative roles of mean and instantaneous dose rate in the effect, especially with electron FLASH.

**Figure 1.**
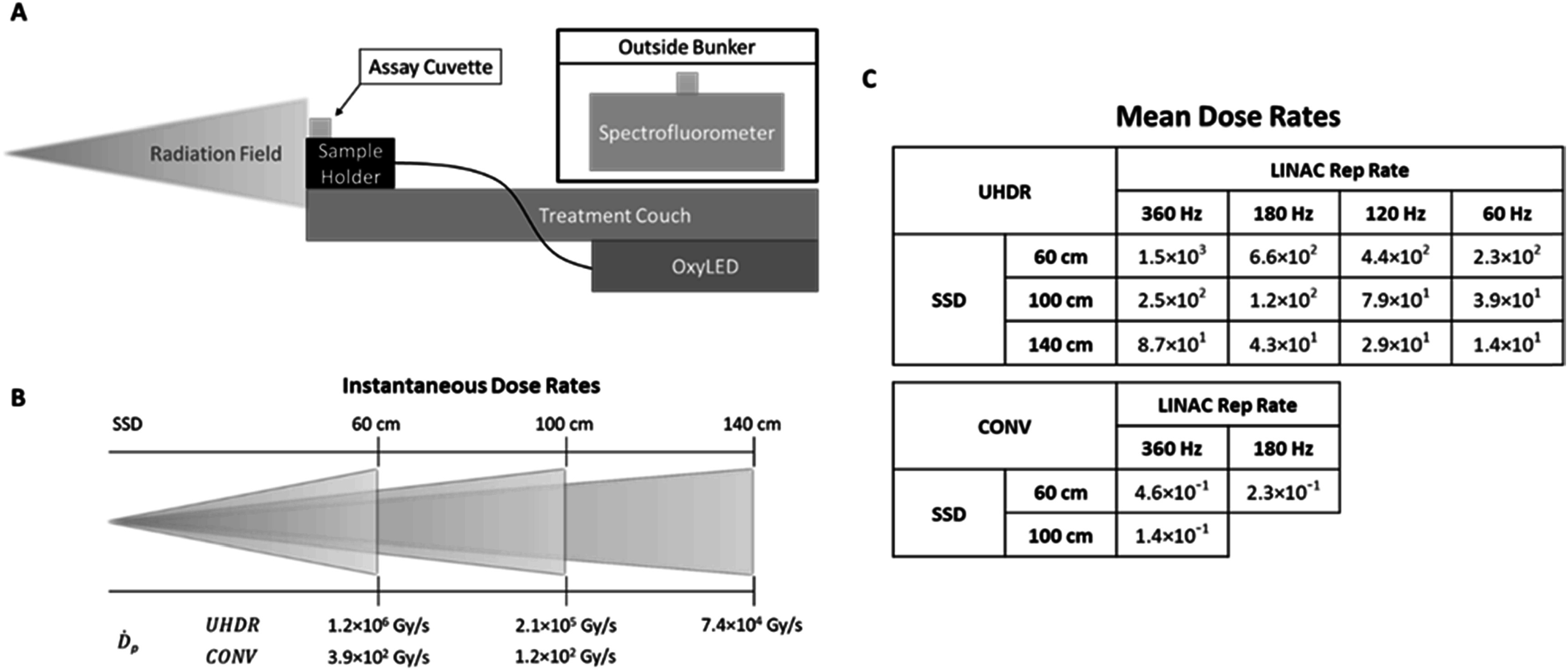
(A) Sample and linac configuration with measurement device placement. (B) Schematic showing corresponding intra-pulse dose rates at each SSD for both UHDR and conventional. (C) Table showing mean dose rates for each SSD and pulse repetition rate. All dose rates are reported in Gy/s.

Independent variation of the mean versus instantaneous dose rates is necessary for a complete understanding of the parameters underlying the FLASH effect, as has been examined in some experimental linacs. In clinical linac treatments, studies have used the source to surface distance (SSD) variation to systematically vary the dose rate (Schuler *et al* 2017), but this also simultaneously varies both instantaneous and mean dose rate. In addition, it is possible to vary the pulse repetition rate of the linac, which alters the mean dose rate, while keeping the instantaneous dose rate fixed. We have used both of these approaches to examine the impact of both instantaneous dose rate and mean dose rate on two parameters that are potentially important in the biological effects of FLASH: H_2_O_2_ yield and ΔpO_2_.

A mechanistic understanding of UHDR radiotherapy effects is needed to allow optimization of the dosimetry choices for FLASH normal tissue sparing effects. While there is very little commonality between all the normal tissues that have been shown to exhibit FLASH sparing (i.e. lung, colon, dermal tissue, brain), one key aspect linking them all is that they are at normal oxygen tensions (typically median value >20 mmHg), whereas tumors are commonly thought to be at lower oxygen levels (typically median value <20 mmHg) (Carreau *et al* 2011, Swartz *et al* 2020, Chen and Gaber 2021). Given the dominance of ROS and the well-known oxygen enhancement ratio in radiation damage, it seems plausible that oxygen is a factor in the differences between normal and tumor tissue sparing from FLASH, even if there is no clear fleshed out mechanism around this today.

## 2. Methods

### 2.1. Electron UHDR delivery and dosimetry

The UHDR beam used in these experiments was a modified Varian Trilogy linac using the same conversion process as described in previous work on a Varian Clinac 2100 C/D (Rahman *et al* 2021). For the duration of single experiment sets, the linear accelerator was reversibly modified so that the x-ray target was held in a retracted position while the carousel was set to an open port, giving access to the pristine 10 MeV electron beam while running in the 10 MV photon mode. For conventional irradiations, the same accelerator was operated in standard 9 MeV electron mode.

For all experiments, a field size of 10 × 10 cm^2^ was used at a gantry angle of 270° and a couch angle of 90°, as shown in figure 1(a), to completely cover the solution samples at each of the source-to-surface distances. Sample cuvettes were placed in a custom 3D printed sample holder with 100% in-fill surrounding the cuvette, allowing for repeatability in set-up conditions across all deliveries and experiments.

Gafchromic Film EBT-XD was used for absolute dosimetry to verify delivered doses to each individual sample. EBT film has been used as a gold standard in UHDR dosimetry due to its dose rate independence (Favaudon *et al* 2014, Jaccard *et al* 2017). Within the context of this work, all film was from a single batch and found to have a variation of less than 1.4% across repeated deliveries. Minor dose reduction was seen in the first 2–4 pulses for all deliveries studied with the linac, but our estimates suggest that this was <5% of the total dose delivered for a 20 Gy sample and that this dose rate reduction would be nearly consistent for all samples. Thus, greater than 95% of the dose delivered was at UHDR values for these experiments, and sample to sample variation in instantaneous and mean dose rate was expected to be low (i.e. <2%).

### 2.2. Dose rate modulation

To evaluate the impact of dose rate more extensively, a wide range of both instantaneous (intra-pulse) and mean (overall) dose rates were employed. Both the mean and instantaneous dose rates were varied by changing the SSD of the sample, between 60 and 140 cm. The mean dose rate was then independently varied at each of the three SSDs by changing the pulse repetition rate of the accelerator. Pulse repetition rate was modified to maintain consistency in time between pulses throughout a delivery. Frequencies of 60, 120, 180 and 360 Hz were used by selecting 100, 200, 300, and 600 MU/min on the linear accelerator.

These spatial and temporal modulations enabled us to investigate 5 instantaneous dose rates and 15 mean dose rates ranging from clinical (conventional) dose rates to UHDRs. Instantaneous and mean dose rates ranged from 10^2^ to 10^6^ Gy s^−1^ and 0.14 to 1500 Gy s^−1^, respectively. Delivered doses ranged from 13 to 42 Gy. Figures 1(b) and (c) summarize all dose rates and configurations used, figure S1 details the average delivered dose at each configuration. Due to differences in MU/min repetition rate options for electron and photon modes, conventional deliveries were limited to 360 and 180 Hz, corresponding to 1000 and 500 MU/min in electron mode, to ensure consistency in time between pulses.

Delivered pulses were counted with a radiation trigger unit (RTU). Using the number of pulses and pulse frequency, total irradiation time was estimated. Mean dose rate was then calculated by dividing the total delivered dose by the total irradiation time. The RTU was also used to collect temporal information of delivered pulses, which showed a pulse duration of 3.5 *μ*s. By dividing total dose by the number of pulses and by the duration of a single pulse, an average instantaneous dose rate was calculated for each individual delivery. A separate work details more information concerning beamline, pulse structure, and conversion methods (Sloop *et al* 2023).

### 2.3. Measurement of ΔpO_2_

Oxyphor PdG4 (or Oxyphor G4) was used as an oxygen probe for this study (Oxygen Enterprises, Philadelphia, PA). PdG4 is a well-characterized oxygen probe (Esipova *et al* 2011) that allows quantification of molecular oxygen across the biological range of pO_2_. Its phosphorescent lifetime varies with oxygen concentration, making it a robust and translatable probe for oximetry.

PdG4 also can be used in protein solutions in a wide range of protein concentrations, i.e. from sub-micromolar to millimolar (Esipova *et al* 2011, Cao *et al* 2021). PdG4 is soluble in water and can be kept in a stock concentration of 200 *μ*M at 10 °C. The stock is then diluted at a 1:100 ratio to 2 *μ*M, to which bovine serum albumin (BSA) is added for the experiments described. In all studies, sample cuvettes were sealed to prevent reoxygenation from occurring.

An Oxyled phosphometer (Oxygen Enterprises, Philadelphia, PA) was used to measure real-time *in vitro* partial pressures of oxygen (pO_2_) prior to, during, and following irradiation at a sampling rate of 50 Hz. Pre-delivery measurements were subtracted from post-delivery measurements to determine the change in pO_2_ due to irradiation. The Oxyled system includes a fiber-coupled LED source pulsed to excite the sample and an avalanche photodiode detector to record the phosphorescent emission of the Oxyphor probe. Using a black cuvette holder, the fiber tips were placed ∼15 mm from the cuvette and shielded from any external changes in ambient light. The phosphometer and recording computer were intentionally placed about 1 m from the radiation field. The system utilized prior calibration of the probe used, with Stern–Volmer fit parameters, to allow extraction of absolute pO_2_ from the lifetime measurements.

Two experiments were conducted using PdG4 to investigate radiation-induced oxygen consumption. The first study investigated the effects of mean and instantaneous dose rates while the second investigated the effect of initial pO_2_. Delivered doses for the dose rate study range from 13 to 42 Gy with a specific accounting of delivered dose by sample in figure S4. The initial pO_2_ study utilized uniform dose steps of approximately 19.5 Gy for conventional dose rate conditions and approximately 21.5 Gy for UHDR conditions with a specific accounting of delivered dose by sample in figure S5.

### 2.4. Amplex red—H_2_O_2_ assay

Amplex Red (Invitrogen, Waltham, MA) is a fluorogenic probe that reacts 1:1 stoichiometry with hydrogen peroxide to form fluorescent resorufin. Due to the short-lived nature of hydroxyl radicals produced during water radiolysis, longer-lived hydrogen peroxide has been used as a proxy for measuring hydroxyl radical yields since it is also a downstream product of radiolysis (Montay-Gruel *et al* 2019). According to previous studies, a concentration of 16.67 *μ*M is optimal for assay use (Montay-Gruel *et al* 2019).

Amplex Red fluorescence data were acquired using the Horiba Fluoromax 4 (Horiba, Kyoto, Kyoto, Japan). Resorufin was excited at 520 nm and the emission was detected at 582 nm. Individual measurements took approximately 30 s. Samples were irradiated in the same sample holder as in the oxygen experiments and measured within 60 s prior to and following irradiation. All measurements were normalized to delivered dose for comparison across many samples.

Calibration was completed by using known amounts of hydrogen peroxide and measuring the fluorescence response until H_2_O_2_ concentration reached a similar magnitude to the probe concentration (16.67 *μ*M), showing a linear relationship between the fluorescence intensity and H_2_O_2_ concentration (figure S2). The assays were tested in two measurement systems, including an Acton 2300 imaging monochromator with a time-gated intensified ICCD from Princeton Instruments (PI-MAX3), and both measurement systems showed linearity of response with H_2_O_2_ generation as a function of dose.

Amplex Red was used to investigate the effect of mean and instantaneous dose rates on radiation induced H_2_O_2_ yield. Delivered doses in this study ranged from 15 to 33 Gy. Figure S6 details specific doses for each sample.

### 2.5. Sample preparation

**Figure 2.**
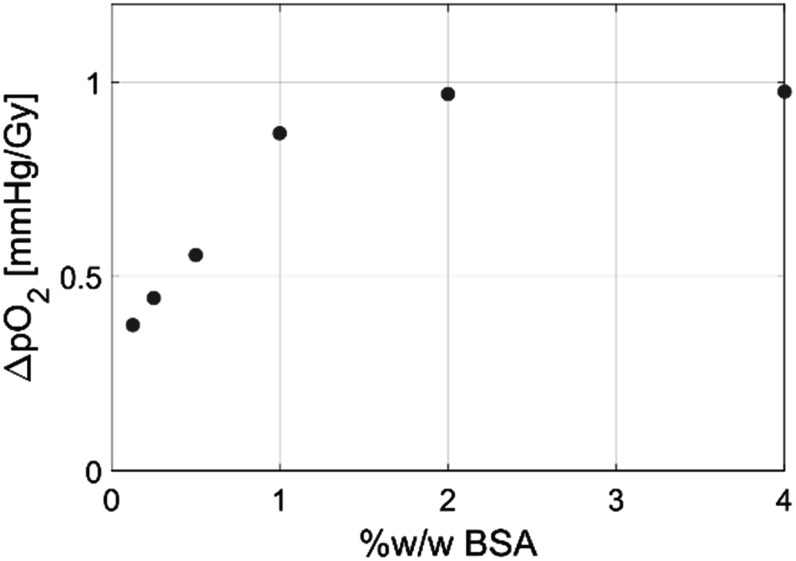
Plot showing dependence of O_2_ consumption per unit dose on BSA concentration at air equilibrated initial pO_2_ (160 mmHg).

All samples were irradiated and measured in 4.5 ml polystyrene cuvettes. Assay solutions were prepared on the day of irradiation and pipetted into the cuvettes within minutes of delivery. BSA was used in these studies to provide a protein microenvironment. Based on a concentration escalation (figure 2) and informed by the physiological albumin concentration of 3%–5% w/w in blood, a concentration of 4% w/w (∼600 *μ*M) was selected to be added to water as the solution base for each of the two assays (Burtis and Ashwood 1998). Furthermore, such a high concentration of BSA compared to that of the oxygen and hydrogen peroxide probes (2 and 16.67 *μ*M) lessens the likelihood of radiation-induced damage to the probes. Radical interactions tend to be non-discriminatory, so a high protein concentration makes radical-protein interactions far more likely than radical-probe interactions, just based upon the probability of nearby interaction from concentration differences.

### 2.6. Statistical analysis

Multivariable regression analysis was employed to investigate the relationship between the logarithm of instantaneous dose rate (x1) and logarithm of mean dose rate (x2), relative to their influence upon the two radiochemical assays. The analysis was performed twice: once with hydrogen peroxide yield per-Gy as the dependent variable and the second with oxygen consumption per-Gy as the dependent variable. Given the mixed use of dose rates, multivariable regression analysis was employed to allow for simultaneous consideration of the two predictors, to understand their individual effects on the assay outcomes. The statistical significance of the coefficients was assessed using the associated *p*-values for each line. The overall significance of the regression model was evaluated using the *F* statistic and significance level.

## 3. Results

### 3.1. Oxygen experiments

**Figure 3.**
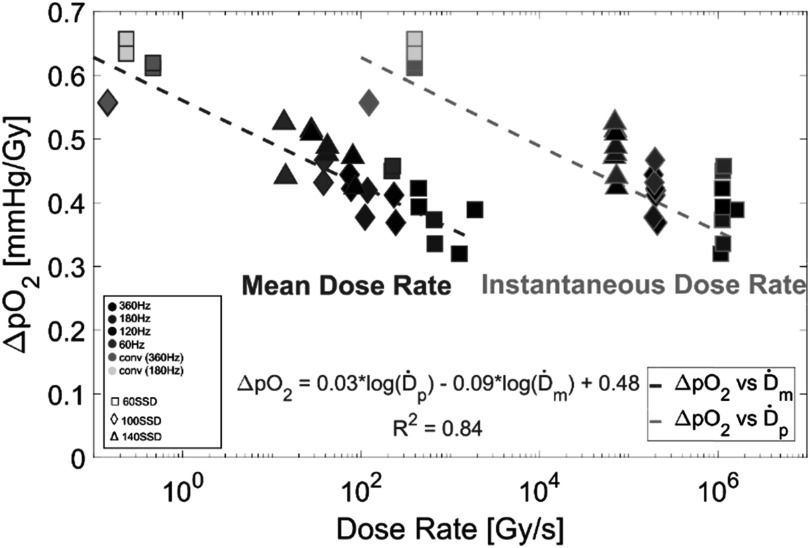
Plot showing dependence of per-Gy O_2_ consumption on both mean dose rate (teal, on left) and instantaneous dose rate (orange, on right). The legend on the left indicates the delivery parameters (pulse repetition frequency and SSD) used to obtain each data point. Lines of best fit are 2D projections of the multivariable regression model where ΔpO_2_ is plotted with respect to mean dose rate and with respect to instantaneous dose rate. The full regression equation is displayed along with the *R* ^2^ value of 0.84. Delivered doses ranged from 13 to 42 Gy.

The effect of UHDR upon *in vitro* oxygen concentration was investigated, as compared to that of conventional dose rates. First, a series of irradiations were delivered while modulating dose rate to establish the dependencies of mean and instantaneous dose rate on per-dose oxygen consumption rate. All samples were oxygenated to air equivalence (160 mmHg) prior to irradiation. The results are graphed in figure 3 and show a dependence of per-Gy O_2_ consumption on both mean and instantaneous dose rates with consumption rate decreasing as dose rate increases. The lowest mean dose rate investigated had a consumption per-Gy ∼1.4 times greater than that of the highest mean dose rate.

**Figure 4.**
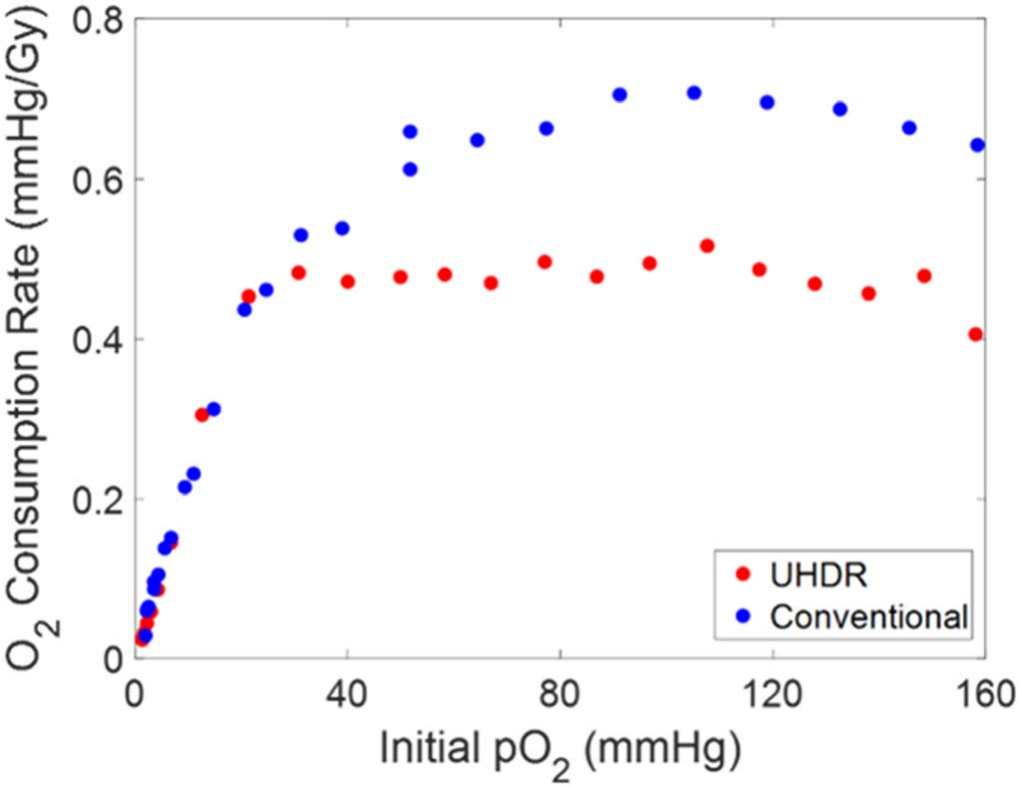
Plot showing effect of initial pO_2_ on O_2_ consumption per unit dose at UHDR (250 Gy s^−1^) in red and conventional dose rate (0.14 Gy s^−1^) in blue. Results acquired at fixed dose levels of 19.5 Gy (conventional) and 21.5 Gy (UHDR).

In a second study, closed samples were progressively irradiated in uniform steps of 19.5 Gy for conventional and approximately 21.5 Gy for UHDR with no more than 30 s of beam-off time between steps and monitored until the samples were completely deoxygenated (0 mmHg). This was performed at 100 cm SSD and 360 Hz under conventional and UHDR conditions to establish the impact of UHDR on the dependence of initial pO_2_ on O_2_ consumption rate. Figure 4 displays the results of this study, showing the dependence of initial pO_2_ on per-dose oxygen consumption for both ultra-high and conventional dose rates at an SSD of 100 cm and a pulse repetition rate of 360 Hz. Figure S5 in the supplemental materials details the exact doses and beam parameters for each delivery.

For initial pO_2_ values above 30 mmHg, observed consumption rates for UHDR delivery are less than conventional delivery. However, initial pO_2_ dependence was observed to be the same below 30 mmHg between the two dose rates.

### 3.2. H_2_O_2_ yield experiments

Radiation-induced changes in H_2_O_2_ concentration were quantified and normalized to delivered dose for a given sample. These generation rates (H_2_O_2_ produced per unit dose) were investigated across many mean and instantaneous dose rates.

**Figure 5.**
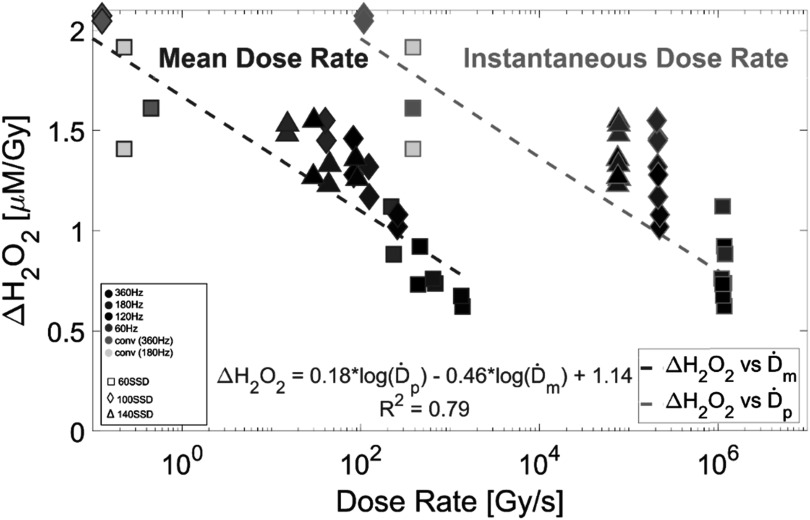
Plot showing dependence of per-Gy H_2_O_2_ yield on both mean dose rate (teal, on left) and instantaneous dose rate (orange, on right). The legend on the left indicates the delivery parameters (pulse repetition frequency and SSD) used to obtain each data point. Lines of best fit are 2D projections of the multivariable regression model where ΔH_2_O_2_ is plotted with respect to mean dose rate and with respect to instantaneous dose rate. The full regression equation is displayed along with the *R* ^2^ value of 0.79. Delivered doses ranged from 15 to 33 Gy.

The data, as shown in figure 5, show a strong dependency of mean dose rate on hydrogen peroxide production per unit dose. The observed yield of H_2_O_2_ decreases as mean dose rate increases. The highest dose rate investigated (1500 Gy s^−1^) showed a per-Gy yield ∼3.3 times less than that of the lowest dose rate (0.14 Gy s^−1^).

### 3.3. Statistical analysis

Multivariable regression analysis showed that the logarithm of the mean dose rate had a statistically significant negative effect upon H_2_O_2_ yield, with a regression coefficient of −0.46 (*p*-value = 0.001), indicating that the yield decreased by 0.46 *μ*M per Gy of dose. In contrast, the logarithm of the instantaneous dose rate did not show a statistically significant relationship with hydrogen peroxide yield per unit dose, as the associated *p*-value = 0.145. However, the overall regression model was statistically significant with an *F* statistic of 52 (*p*-value = 5.4 × 10^−10^), suggesting that the model which combines mean dose rate and instantaneous dose rate information explains a significant portion of the variance in the H_2_O_2_ yield. These findings indicate that mean dose rate is a significant predictor of hydrogen peroxide yield per unit dose, for the range of values studied here. Furthermore, this also indicates that the inclusion of instantaneous dose rate as a predictor does not significantly improve the regression model.

Similarly, for the oxygen consumption per unit dose, the multivariable regression analysis showed that the logarithm of the mean dose rate had a statistically significant negative effect, with a coefficient of −0.094 (*p*-value = 0.0009), indicating ΔpO_2_ per dose decreased by 0.094 mmHg Gy^−1^. In contrast, the logarithm of the instantaneous dose rate did not show a statistically significant relationship with change in pO_2_ per unit dose, with *p*-value = 0.25. However, the overall regression model was statistically significant with an *F* statistic of 70 (*p*-value = 2.1 × 10^−11^), suggesting that the model explains a significant portion of the variance in the oxygen consumption per unit dose. Just as with H_2_O_2_ yield, these findings indicate that mean dose rate is a more important predictor of oxygen consumption per unit dose compared to instantaneous dose rate, for the range of values studied here.

**Figure 6.**
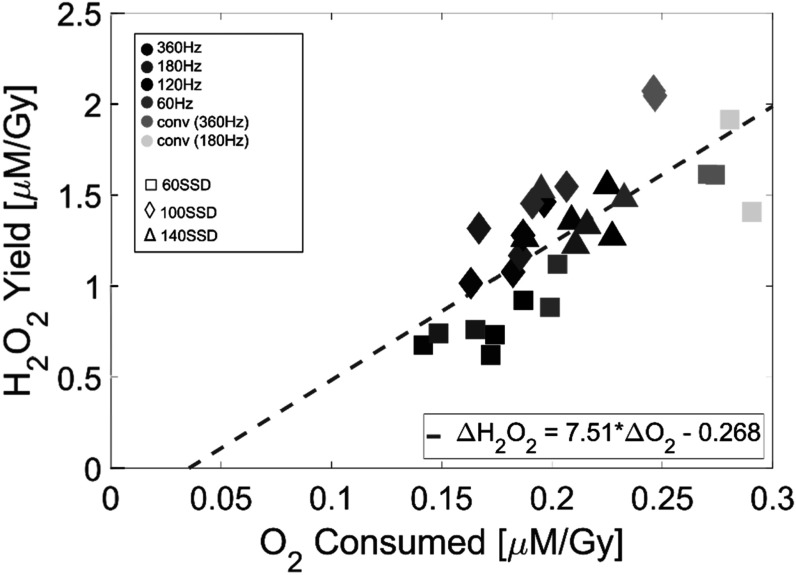
Plot showing the relationship between observed O_2_ consumption and H_2_O_2_ yield. Data was paired by set-up condition. The regression fit is included (*R* ^2^ = 0.58) along with the same data point legend as found in figures 3 and 5. Delivered doses ranged from 13 to 42 Gy.

Furthermore, a regression analysis was performed on the hydrogen peroxide and oxygen data by pairing the data points based on dose rate condition (figure 6). The analysis showed that consumed oxygen per unit dose is a significant predictor of hydrogen peroxide yield per unit dose with a coefficient of 7.51 (*p*-value = 1.98 × 10^−6^), indicating that 7.51 micromole of H_2_O_2_ is yielded for every micromole of O_2_ consumed. The exact radiochemical explanation and implicaiton of this relationship is undetermined at this time, although they are expected to be related.

The qualifications for these interpretations are specifically that only 5 instantaneous dose rates were investigated and only two parameters were used for analysis, so the implications with respect to the FLASH effect of these findings are not concrete or final. Mean dose rate and instantaneous dose rate were chosen as variables since they were the only parameters explicitly adjusted in the experiments. It is possible that other beam parameters, including total delivery time, pulse repetition frequency, and pulse width, may contribute to the observed radiochemical phenomena as well, but these remain to be tested in future work. Additionally, the chemical measurements were not acquired simultaneously. As such, further research where more dose rates are used and in which dose rate types are varied independently and hydrogen peroxide and oxygen are measured in the same sample are needed to better understand the complex relationships between dose rate parameters of UHDR and the underlying mechanisms driving these radiochemical effects.

## 4. Discussion

The focus of this study was on using different mean and per-pulse instantaneous dose rates with a broad electron beam from a converted clinical linac. Previously, systematic studies of these dose rate factors have limited the conclusions that can be drawn in FLASH UHDR studies, because it can be challenging to vary these parameters independently on a converted linac. Through variation in pulse frequency, affecting mean dose rate only, and variation in SSD location, affecting both mean and instantaneous dose rates, we were able to statistically assess the relative effects of these two factors. It is possible that total delivery time, which increased as number of pulses increased or pulse frequency decreased, has a unique impact on these phenomena, but this remains to be tested further in separate work.

The results of this study are important because they may help determine how to optimize FLASH dose delivery. The fact that oxygen is consumed during radiotherapy has been known for a long time, but just recently demonstrated *in vivo* (El Khatib *et al* 2022). Additionally, an expected dependence upon BSA concentration was noted with UHDR protons (El Khatib *et al* 2022) and confirmed here with UHDR electrons (figure 2). The use of BSA as a dominant protein provides a first order match to *in vivo* testing, where albumin is the most dominant protein in the plasma. Still, it is possible, and perhaps likely that further interactions with the protein and lipid milieu present *in vivo* are even more complex than seen here (Van Slyke *et al* 2022). Intermediate steps towards *in vitro* or *in vivo* studies could utilize protein mixtures that approach *in vivo*. Still, the use of high concentration BSA provides a microenvironment that is closer to *in vivo* than seen with simpler pure water tests due primarily to the abundant presence of proteins *in vivo*, which act as targets for generated radical species. The presence of organic molecules contributes to radiation-induced chemical yield and consumption pathways due to their interactions with radical species (Hirayama *et al* 2013, Favaudon *et al* 2022).

The oxygen consumption versus dose rates is shown and illustrates that increasing mean dose rate has a significant effect upon reducing the oxygen consumption seen for doses between 13 and 42 Gy (figure 3). This study is perhaps the most important observation of this work, illustrating a key component of ROS production in UHDR irradiation, which may be unique. The relationship between this and the *in vivo* FLASH effect is still unknown, but strong changes in ROS seem plausibly related to the effects seen *in vivo* from FLASH.

Furthermore, the initial pO_2_ study showed a discrepancy between conventional and UHDR in the plateau region above 30 mmHg, but notable agreement in the linear region below 30 mmHg. *In vivo*, there is a wide range of oxygen tensions that measured in normal tissues and values depend a lot on the tissue type going all the way from low oxygenated tissue such as superficial skin (8–11 mmHg) up to intermediate oxygenated tissues such as muscle, brain (30–50 mmHg) up to highly oxygenated such as intestine (50–60 mmHg) kidney cortex (50–80 mmHg) (Carreau *et al* 2011, McKeown 2014). Because of this further, *in vivo* oxygen studies might be conducted to determine how initial pO_2_ contributes to the biological FLASH effect.

The second half of this work concerns the generation of a key ROS species in H_2_O_2_ production and how changes to instantaneous and mean dose rates spanning from conventional to UHDR affect the generation of H_2_O_2_. It was found that increasing mean dose rate results in a significant decrease in hydrogen peroxide produced per-Gy delivered (figure 5), just as was seen in the oxygen consumption experiments (figure 3). This finding reinforces the assertion that there is a significant dependence on dose rate for these radiochemical phenomena, which may be related to the FLASH effect.

This is not the first time Amplex Red has been used to quantify generated hydrogen peroxide to understand the radiochemical differences between conventional and UHDRs. Previously, Montay-Gruel *et al* reported a statistically significant decrease in H_2_O_2_ generation for FLASH delivery compared to equivalent doses of conventional delivery in pure water samples (Montay-Gruel *et al* 2019). This work, however, is the first time that aqueous protein solutions have been irradiated at similar doses across a broad range of dose rates with specific attention given to dose rate type (instantaneous compared to mean) in addition to whether a particular delivery falls into the conventional or UHDR range.

The implications of these findings are significant to understanding the underlying mechanisms of the FLASH effect in addition to the parametric considerations of UHDR delivery. Hydroxyl radicals formed through water radiolysis play a significant role in radiation-induced DNA damage pathways (Blain *et al* 2022, Van Slyke *et al* 2022). Interactions of these hydroxyl radicals with DNA can cause single- and double-strand breaks, which may lead to cellular and tissue damage. Previously, H_2_O_2_ measurements have been used as an indicator for other ROS, including hydroxyl radicals (Montay-Gruel *et al* 2019). We observed that, as mean dose rate increases, H_2_O_2_ generated per unit dose decreases significantly. While the exact mechanism for decreased ROS yield is presently unknown, recombination of radicals due to a high local concentration from large depositions of dose in short deliveries has become a popular theory (Favaudon *et al* 2022). It is, however, important to note that the dose rate experiments presented in this work were all performed with samples at air-equilibrated oxygen tension. As shown by figure 4, sub-30 mmHg initial pO_2_ results in less oxygen consumed per unit dose, therefore the magnitude of change to these chemical phenomena at higher dose rates likely differs at physiologic and hypoxic oxygen levels found *in vivo*. Though these findings are limited to *in vitro* protein solutions, it is possible that a similar phenomenon occurs *in vivo* wherein UHDR delivery induces a lower H_2_O_2_ yield than conventional irradiation, thereby contributing to the observed sparing effect.

Upon direct comparison of the results from the oxygen and hydrogen peroxide studies, a statistically significant relationsip was found between oxygen consumed and hydrogen peroxide yield per unit dose. Interpretation of this finding is limited at this time, but the reduction in both as dose rate increases may have implications with respect to the FLASH effect specifically related to oxygen consumption being an indicator of ROS production. Further experiments where these species are simultaneously measured in a combined assay are necessary to fully unravel the correlation.

## 5. Conclusion

This work was a systematic study of the effect of dose rate on both oxygen consumption and hydrogen peroxide yield per unit dose *in vitro* for mean and instantaneous dose rates ranging from conventional to UHDR. It was found that mean dose rate has a statistically significant impact on the amount of O_2_ consumed and the amount of H_2_O_2_ produced per-Gy during irradiation. As mean dose rate increases, significant reductions in the per-Gy rates at which oxygen is consumed and hydrogen peroxide is generated were observed *in vitro*.

The results indicate that mean dose rate may have a dominant effect over instantaneous dose rate on the outcome of the two radiochemical phenomena investigated. However, the extent to which intantaneous dose rate was varied in this work was limited. More extensive studies in which the mean or instantaneous dose rate is fixed while the other is modulated for many fixed values should be conducted if possible. Such a study would allow for stronger conclusions to be drawn on the unique impact of each dose rate type, but this work suggests that the impacts of the mean dose rate on the phenomena discussed is stronger.

Additionally, oxygen consumption per-Gy was found to be dependent on initial pO_2_ for both conventional and UHDR electron delivery *in vitro*. Under both conditions, oxygen consumption decreased as initial pO_2_ decreased. Interestingly, UHDR and conventional are notably similar in initial pO_2_ dependence at levels <30 mmHg, so implications on the FLASH effect remain unclear as both tumors and healthy tissues have a wide range of physiological pO_2_ levels.

## Data Availability

All relevant research data is available from the corresponding author upon reasonable request. The data that support the findings of this study are available upon reasonable request from the authors.

## Funding

The authors acknowledge funding from the National Cancer Institute through the Dartmouth Cancer Center P30 CA023108 and the Carbone Cancer Center P30 CA014520 and through contract U01 CA260446.
